# Should manual detorsion be a routine part of treatment in testicular torsion?

**DOI:** 10.1186/s12894-017-0276-5

**Published:** 2017-09-15

**Authors:** Arif Demirbas, Demirhan Orsan Demir, Erim Ersoy, Mucahit Kabar, Serkan Ozcan, Mehmet Ali Karagoz, Ozgecan Demirbas, Omer Gokhan Doluoglu

**Affiliations:** 1 Department of Urology, Ankara Training and Research Hospital, 06340, Sukriye, Altındağ Ankara, Turkey; 2 Department of Pediatrics, Ankara Dr. Sami Ulus Women Health, Children’s Training and Research Hospital, 06340 Ankara, Turkey

**Keywords:** Testicular torsion, Manual detorsion, Orchiopexy, Orchiectomy

## Abstract

**Background:**

It was aimed to investigate the efficiency and reliability of the manual detorsion (MD) procedure in patients diagnosed with testicular torsion (TT).

**Methods:**

A retrospective analysis was made of the data of 57 patients diagnosed with TT, comprising 20 patients with successful MD (Group I), 28 patients who underwent emergency orchiopexy (Group II), and 9 patients applied with orchiectomy (Group III). The groups were compared in respect of age, and duration of pain. The success rate of MD, the time of testicular fixation (TF), any problems encountered in follow-up, and follow-up times were analyzed in Group I. Data were analyzed with P-P pilot, Mann-Whitney U, Kruskal Wallis and Chi-square tests. A value of *p* < 0.05 was considered statistically significant.

**Results:**

MD was successful and detorsion could be achieved in 20 of 26 patients. The groups were similar in respect of age (*p* = 0.217). The median duration of pain was 3 (1–8), 4 (1–72), and 48 (12–144) hours in Groups I, II, and III, respectively, and determined as similar in Groups I and II (*p* = 0.257), although a statistically significant difference was determined between the 3 groups (*p* < 0.001). TF was applied to Group I after median 10 (0–45) days, and no parenchymal disorder was determined in the median follow-up period of 21.5 (2–40) months.

**Conclusion:**

MD that can be easily and immediately performed after the diagnosis of TT decreases ischemia time. This seems to be an efficient and reliable procedure when applied together with elective orchiopexy, as a part of the treatment.

**Electronic supplementary material:**

The online version of this article (10.1186/s12894-017-0276-5) contains supplementary material, which is available to authorized users.

## Background

Testicular (spermatic cord) torsion (TT), which was first described by Hunter, is an emergency urological diagnosis that results in ischemic organ injury in the affected testis, and requires urgent diagnosis and treatment [1, 2]. Cold weather, activation of the cremasteric reflex, trauma, undescended testis, and fast enlargement of testis during puberty are the known risk factors in susceptible individuals. It shows a bimodal distribution, and peaks in the neonatal period and puberty. Prevalence has been reported as 8.6/ 100,000 between the ages of 10 and 19 years in the United States [1–4].

With the exception of neonates, patients usually present with unilateral severe pain that has been present for a few hours [5, 6]. Pain is rarely mild, and it radiates to the inguinal region and abdomen. The most frequent physical examination findings are testicular tenderness and loss of the cremasteric reflex [1, 7]. An abnormal position of the testis is more common than other causes for acute scrotal pain [8]. Doppler ultrasonography (USG), scintigraphy, dynamic magnetic resonance (MR), and high-resolution USG are the diagnostic imaging modalities [3].

The most important factors for testicular salvage before it atrophies are known to be the duration between the onset of symptoms and detorsion, and the degree of the torsion [9, 10]. Emergency surgery after the diagnosis aims to shorten the duration of ischemia [1, 2, 11]. Manual detorsion (MD), as described by Nash, has been suggested before surgery to return blood flow faster, and some authors have indicated it as an alternative to surgery [1, 12, 13].

In this study, it was aimed to investigate the efficiency of MD, whether it could be a routine part of treatment or an alternative to surgery in patients with TT due to time saved by the application, and the reliability of elective orchiopexy rather than emergency orchiopexy in the light of information provided in literature.

## Methods

A retrospective analysis was made of a total of 57 patients admitted to our outpatient clinic or Emergency Department between 2011 and 2015 with acute scrotal pain, and who were diagnosed with TT based on physical examination findings and decreased or absent testicular blood flow on Doppler USG. The approval of the Local Ethics Committee was obtained before starting the study. The MD procedure was applied by three urologists in our clinic, and it was not performed by other urologists. Group I comprised 20 patients with successful MD, Group II comprised 28 patients who underwent emergency orchiopexy or orchiopexy after failure of MD, and Group III comprised 9 patients that had emergency scrotal exploration and orchiectomy. The patients in Group I had bilateral testicular fixation (TF) under elective conditions after MD, and the patients in Group II had bilateral TF during emergency orchiopexy.

The groups were compared in respect of age, and the time from onset of scrotal pain to admission to hospital (duration of pain). In Group I, the time of elective TF performed after MD and the number of the patients with a successful MD procedure were determined. In addition, the follow-up period, and the presence of testicular atrophy findings on Doppler USG were analyzed in Group I. The patients that had orchiopexy and orchiectomy (Groups II and III) were analyzed in respect of the time from onset of pain to the start of surgery in order to determine the gain in ischemia duration with MD compared to emergency scrotal exploration.

MD was performed without using any anesthesia technique to preserve the feeling of pain, in consideration of retorsion risk in the affected testis. The affected testis was rotated laterally for detorsion. However, when lateral rotation was not successful due to lateral TT, then medial rotation was applied [14]. The success of MD was defined as the immediate relief of symptoms and improvement of the physical examination findings, and the success was confirmed by normal testicular arterial and venous blood flow on Doppler USG, which was performed immediately after MD.

Patients in Group I and Group II were followed up with monthly physical examinations for the first 6 months after diagnosis and Doppler USG was applied at 3-month intervals. Subsequent follow-up was physical examinations at 6-month intervals.

The study was conducted in accordance with the principles of Declaration of Helsinki 2008.

### Statistical analysis

The statistical analysis of data was performed using SPSS version 15.0 (SPSS Inc., Chicago, IL, United States) statistics software. Conformity to normal distribution of the data was analyzed with histogram and P-P pilot test. The Mann-Whitney U and Kruskal Wallis tests were used to compare continuous variables that did not show normal distribution. The Chi-square test was used to compare categorical variables. A value of *p* < 0.05 was considered statistically significant.

## Results

MD was performed in 26 patients diagnosed with TT. Successful MD was recorded in 20 (76%) patients, and 6 (24%) patients had emergency orchiopexy due to failure of MD. The ages of the patients were similar in all 3 groups (*p* = 0.217). The median duration of pain was 3 (1–8) hours in Group I, 4 (1–72) hours in Group II, and 48 (12–144) hours in Group III, with a significant difference determined between the groups (*p* < 0.001) (Table 1). The comparison of Groups I and II where testicular salvage could be achieved revealed that those groups were similar in respect of duration of pain (*p* = 0.257) (Table 2).

**Table 1:** The symptoms on admission in patients diagnosed with testicular torsion

	Group I *n* = 20	Group II *n* = 28	Group III *n* = 9	p
Age	17.5	19.5	20	= 0.217
Duration of pain (hours, min-max)	3, 1–8	4, 1–72	48, 12–144	<0.001

**Table 2:** Comparison of duration of pain in the groups that had testicular salvage

	Group I *n* = 20	Group II *n* = 28	p
Duration of pain (hours, min-max)	3, 1–8	4, 1–72	= 0.257

TF was performed under elective conditions at median 10 (0–45) days after MD in 18 of 20 patients. The other 2 patients, aged 45 and 36 years, respectively, were followed up without TF, as they did not accept the procedure. In the follow-up of these 2 patients, no painful episodes developed and no pathological findings were determined on physical examination or on Doppler USG. During the median follow-up period of 21.5 (2–40) months, none of the patients in Group I had atrophy or parenchymal disorders on physical examination or Doppler USG after MD and TF. No painful episode developed in any patient in the period up to elective orchiopexy. In the 6 patients applied with emergency orchiopexy due to unsuccessful MD, no testicular atrophy or other pathological findings were encountered. The median ischemia durations between diagnosis and scrotal exploration were 90 (20–240) and 80 (45–180) minutes in Groups II and III, respectively (*p* = 0.636) (Table 3).

**Table 3:** The time between diagnosis and scrotal exploration in patients that had orchiopexy or orchiectomy

	Group II *n* = 28	Group III *n* = 9	p
The time between diagnosis and exploration (minutes, min-max)	90, 20–240	80, 45–180	0.636

## Discussion

TT is regarded as a race against time, and the histological changes of testicular injury appear in hours, and even in minutes [1–3]. It has been reported that irreversible ischemic changes occur 4–6 h after ischemic scrotum, and that 80% of patients need orchiectomy due to necrosis after 24 h if the testis is not detorsioned [3, 14, 15]. Studies have also shown that the duration between onset of symptoms and treatment caused disturbances in semen quality through autoimmune mechanisms [16].

Emergency scrotal exploration has been recommended as the standard treatment method for derotation of the rotated spermatic cord, and restoration of testicular blood flow [1–3]. However, minutes and even hours may be needed to prepare for this emergency procedure. Our five-year experience showed there was a significant time loss between diagnosis and treatment (80–90 min, Table 3) and this was reflected in the ischemia time.

MD was first described in 1893. It was performed to restore blood flow and give rapid pain relief. The efficiency of the procedure has been investgated by various authors [17–20]. Catolica [18] published the largest series with 34 patients, and reported that MD was successful in all patients, TF was performed under elective conditions in 6 patients, and the duration between urological consultation and surgery was between 1 h and 2 months in the 34 patients included in the study. The author also reported that retorsion was not evident in any of the patients, the ischemic testis was saved, and an emergency procedure became an elective procedure. A study from the Netherlands reported MD in 17 patients, 14 of which were successful, orchiopexy was performed electively (waiting time was mean 12 h, ranging from 2 h to 3 months), retorsion was not seen in any of the patients, and none of the patients had testicular atrophy after a mean follow-up period of 22 months. The procedure failed in 3 patients, and excessive scrotal edema and pain were considered to be responsible for this since no anesthesia was used to be able to determine “sudden relief of pain” that was used as the success criterion [20]. In a study by Sessions et al. [14], residual torsion was determined during orchiopexy in 17/53 (38%) patients who had been applied with manual detorsion and it was concluded that orchiopexy should not be delayed. However, as there are no studies of high level evidence on this subject in literature, whether orchiopexy should be applied electively or not following manual detorsion does not come under the heading of a recommendation.

In the current series of 26 patients, MD was successful in 20 (76%) patients at the time of diagnosis, and it was determined that testicular salvage was achieved after long term follow-up (median: 21.5 months). Attempting MD and success after the procedure made a significant time-saving compared to orchiopexy in respect of testicular ischaemia time (median: 90 min). In the development of testicular damage, as it is known that every minute is valuable, the median ischaemia-free time of 90 mins gained with MD, suggests that it is important in the treatment of TT [1–3]. Elective TF and the absence of any retorsion in this waiting period can be considered to show the safety of MD. However, even if it is wanted to apply TF immediately after MD, it can be considered necessary to apply it at the time of MD diagnosis. Even after diagnosis, the time of transporting the patient to the operating room and preoperative preparation means ‘a period with ischaemia’. A comparison was made of the patients that had MD and those with emergency orchiopexy (Group II) in order to determine the effect of the duration of torsion on the success of MD and no effect was observed.

## Conclusion

MD is a non-invasive procedure, may be applied as soon as the diagnosis is made, decreases the duration of ischemia when compared to emergency scrotal exploration, and the long-termresults of this study have shown that it is safe when applied together with elective orchiopexy. MD is a simple procedure that may be performed safely with little to no delay in definite scrotal exploration and orchiopexy, which remains the gold standard.

## Additional file

Additional file 1: Including SPSS data of this article in 5 page which has been converted to word format. (RTF 254 kb)

## Abbreviations

MD: Manual detorsion
TF: Testicular fixation
TT: Testicular torsion
