# Supplementary material for: Should manual detorsion be a routine part of treatment in testicular torsion?
Source: BMC Urol. 2017 Sep 15;17:84. doi: 10.1186/s12894-017-0276-5 (PMC5602835; doi:10.1186/s12894-017-0276-5)
Supplement: Additional file 1: — Including SPSS data of this article in 5 page which has been converted to word format. (RTF 254 kb) [file 12894_2017_276_MOESM1_ESM.rtf]

USE ALL.
COMPUTE filter_$=(Groups = 1).
VARIABLE LABEL filter_$ 'Groups = 1 (FILTER)'.
VALUE LABELS filter_$  0 'Not Selected' 1 'Selected'.
FORMAT filter_$ (f1.0).
FILTER BY filter_$.
EXECUTE .
FREQUENCIES
  VARIABLES= AGE DURATIONOFPAIN Thetimebetweendiagnosisandexploration TFafterMD
  /STATISTICS=STDDEV MINIMUM MAXIMUM MEAN MEDIAN
  /ORDER=  ANALYSIS .

Frequencies

GROUP 1

	Statistics

 	AGE	DURATION OF PAIN(HOURS)	The time between diagnosis and exploration (minutes)	TF after MD (DAY)	
N	Valid	20	20	0	18	
 	Missing	0	0	20	2	
Mean	20,2000	3,3500	 	11,1667	
Median	17,5000	3,0000	 	10,0000	
Std. Deviation	8,35149	1,59852	 	11,49552	
Minimum	13,00	1,00	 	,00	
Maximum	45,00	8,00	 	45,00	


USE ALL.
COMPUTE filter_$=(GROUPS = 2).
VARIABLE LABEL filter_$ 'GROUPS = 2 (FILTER)'.
VALUE LABELS filter_$  0 'Not Selected' 1 'Selected'.
FORMAT filter_$ (f1.0).
FILTER BY filter_$.
EXECUTE .
FREQUENCIE
  VARIABLES=AGE DURATIONOFPAIN Thetimebetweendiagnosisandexploration TFafterMD
  /STATISTICS=STDDEV MINIMUM MAXIMUM MEAN MEDIAN
  /ORDER=  ANALYSIS .

Frequencies

GROUP 2

	Statistics

 	AGE	DURATION OF PAIN(HOURS)	The time between diagnosis and exploration (minutes)	TF after MD (DAY)	
N	Valid	28	28	26	0	
 	Missing	0	0	2	28	
Mean	21,2500	8,5357	89,5000	 	
Median	19,5000	4,0000	90,0000	 	
Std. Deviation	7,13170	15,37068	48,65162	 	
Minimum	9,00	1,00	20,00	 	
Maximum	43,00	72,00	240,00	 	


USE ALL.
COMPUTE filter_$=(GRUPLAR = 3).
VARIABLE LABEL filter_$ 'GRUPLAR = 3 (FILTER)'.
VALUE LABELS filter_$  0 'Not Selected' 1 'Selected'.
FORMAT filter_$ (f1.0).
FILTER BY filter_$.
EXECUTE .
FREQUENCIES
  VARIABLES= AGE DURATIONOFPAIN Thetimebetweendiagnosisandexploration TFafterMD
  /STATISTICS=STDDEV MINIMUM MAXIMUM MEAN MEDIAN
  /ORDER=  ANALYSIS .

Frequencies

GROUP 3

	Statistics

 	AGE	DURATION OF PAIN(HOURS)	The time between diagnosis and exploration (minutes)	TF after MD (DAY)	
N	Valid	9	9	9	0	
 	Missing	0	0	0	9	
Mean	23,5556	65,1111	85,5556	 	
Median	20,0000	48,0000	80,0000	 	
Std. Deviation	8,20230	50,92260	38,19722	 	
Minimum	16,00	12,00	45,00	 	
Maximum	43,00	144,00	180,00	 	


FILTER OFF.
USE ALL.
EXECUTE .
NPAR TESTS
  /K-W= AGE DURATIONOFPAIN Thetimebetweendiagnosisandexploration TFafterMD   BY
  GROUPS(1 3)
  /MISSING ANALYSIS.

NPar Tests

[DataSet1] F:\MANUEL DETORSION\TORSION..sav

Kruskal-Wallis Test

	Ranks

 	GROUPS	N	Mean Rank	
AGE	MANUEL DETORSION	20	24,53	
 	SKROTALEXPLORATION	28	30,04	
 	ORCHİECTOMY	9	35,72	
 	Total	57	 	
DURATION OF PAIN (HOURS)	MANUEL DETORSION	20	21,83	
 	SKROTALEXPLORATION	28	26,84	
 	ORCHİECTOMY	9	51,67	
 	Total	57	 	
Thetimebetweendiagnosisandexploration (minutes)	SKROTALEXPLORATION	26	18,48	
 	ORCHİECTOMY	9	16,61	
 	Total	35	 	
TF after MD (DAY)	MANUEL DETORSÝYON	18	9,50	
	Total	18(a)	 	
a  There is only one non-empty group. Kruskal-Wallis Test cannot be performed.


	Test Statistics(a,b)

 	AGE	DURATION OF PAIN(HOURS)	The time between diagnosis and exploration (minutes)	
Chi-Square	3,059	21,333	,224	
df	2	2	1	
Asymp. Sig.	,217	,000	,636	
a  Kruskal Wallis Test
b  Grouping Variable: GROUPS


NPAR TESTS
  /M-W= AGE DURATIONOFPAIN Thetimebetweendiagnosisandexploration  BY
  GROUPS(1 2)
  /MISSING ANALYSIS.

NPar Tests

GROUP 1-2

Mann-Whitney Test

	Ranks

 	GROUPS	N	Mean Rank	Sum of Ranks	
AGE	MANUEL DETORSION	20	21,85	437,00	
 	SKROTALEXPLORATION	28	26,39	739,00	
 	Total	48	 	 	
DURATION OF PAIN (HOURS	MANUEL DETORSION	20	21,83	436,50	
 	SKROTALEXPLORATION	28	26,41	739,50	
 	Total	48	 	 	
Thetimebetweendiagnosisandexploration (minutes)	MANUEL DETORSION	0(a)	,00	,00	
 	SKROTALEXPLORATION	26	13,50	351,00	
 	Total	26	 	 	
TF after MD (DAY)	MANUEL DETORSION	18	9,50	171,00	
 	SKROTALEXPLORATION	0(a)	,00	,00	
 	Total	18	 	 	
a  Mann-Whitney Test cannot be performed on empty groups.


	Test Statistics(a)

 	AGE	DURATION OF PAIN(HOURS)	
Mann-Whitney U	227,000	226,500	
Wilcoxon W	437,000	436,500	
Z	-1,113	-1,134	
Asymp. Sig. (2-tailed)	,266	,257	
a  Grouping Variable: GROUPS


  GROUPS(2 3)
  /MISSING ANALYSIS.

NPar Tests

[GROUP 2-3]

	


Mann-Whitney Test

	Ranks

 	GROUPS	N	Mean Rank	Sum of Ranks	
AGE
 
 	SKROTALEXPLORATION	28	18,14	508,00	
 	ORCHİECTOMY	9	21,67	195,00	
 	Total	37	 	 	
DURATION OF PAIN (HOURS)
 
 	SKROTALEXPLORATION	28	14,93	418,00	
 	ORCHİECTOMY	9	31,67	285,00	
 	Total	37	 	 	
Thetimebetweendiagnosisandexploration (minutes)	SKROTALEXPLORATION	26	18,48	480,50	
 	ORCHİECTOMY	9	16,61	149,50	
 	Total	35	 	 	


	Test Statistics(b)

 	AGE	DURATION OF PAIN(HOURS)	The time between diagnosis and exploration (minutes)	
Mann-Whitney U	102,000	12,000	104,500	
Wilcoxon W	508,000	418,000	149,500	
Z	-,853	-4,050	-,474	
Asymp. Sig. (2-tailed)	,394	,000	,636	
a  Not corrected for ties.
b  Grouping Variable: GROUPS


	
